# Myofibroepithelial Polyp of the Ureter

**Published:** 2014-05-21

**Authors:** Dilip Kumar Pal, Kaushik Sarkar, Debasis Chakrabortty

**Affiliations:** 1 Dept. of Urology, Institute of Post Graduate Medical Education and Research, Kolkata-700006, India;; 2 Dept. of Pathology, Institute of Post Graduate Medical Education and Research, Kolkata-700006, India;

**Keywords:** Ureteral tumors, Fibroepithelial polyp, Myofibroepithelial polyp, Ureteroscopy

## Abstract

Ureteral tumors are rare and benign tumors are even rarer. Most of such tumors are diagnosed after nephroureterectomy assuming a malignant lesion, but with modern technological advancements like contrast enhanced CT scan or MR urography and ureteroscopic biopsy, benign nature of such tumors can be established preoperatively with an aim to preserve the renal unit. Here we report a case of 10-year old boy who presented with chronic right loin pain. DTPA scan showed complete loss of function of the right kidney. He was diagnosed to have an inflammatory myofibroepithelial polyp of right lower ureter and treated by nephroureterectomy.

## INTRODUCTION

Primary ureteral tumors constitute only 1% of all upper genitourinary tract neoplasms.[1] Benign ureteral lesions are rarer and mostly of mesodermal origin.[2] Only 140 cases of fibroepithelial polyps are recorded in English literature.[3] But till now no case of myofibroepithelial polyp has been documented. We herein report a case of benign myofibroepithelial polyp of right ureter in a child.

## CASE REPORT

A 10-year-old boy presented with off and on right loin pain for the last three years. He had dysuria with few episodes of hematuria over last six months with passage of fleshy mass per urethra prior to admission.There was no history of trauma or fever. On examinationhis right renal angle was full and the right kidney was just palpable. His hemoglobin level was 9.8gm% with neutrophilia. Renal biochemical parameters were within normal limits. Urine routine and microscopic examination showed plenty of pus cells with few RBC. Ultrasonography of abdomen showed right sided gross hydroureteronephrosis with ill-defined space occupying lesion in the right lower ureter. MR Urography suggested a gross dilatation of the right kidney and ureter with a polypoid lesion in the lower ureter. A DTPA renogram revealed complete loss of function in the right kidney with no evidence of perfusion and a normal excretion curve on the left side. Ureteroscopy showed a smooth surface polyp in the right lower ureter with complete obstruction of the lumen through which a guide wire could not be negotiated. Ureteroscopic biopsy suggested a polyp with degenerative changes without any evidenceof malignancy. A right sided nephroureterectomy was undertaken as there was no function in that kidney. On longitudinal cut section of the ureter, a smooth polyp of 1cm x 1.5cm was found 2cm above the vesicoureteric junction (Fig.1). Histopathological examination showed smooth muscles with inflammatory cells and perivascular histiocytes in a fibrovascular stroma clothed by transitional epithelium (Fig.2).The lining epithelium of the polyp merged with the urothelium of the ureter. On immunohistochemistry the tumor cells expressed Alk-1 suggesting inflammatory myofibroblastic tumor.

**Figure:**
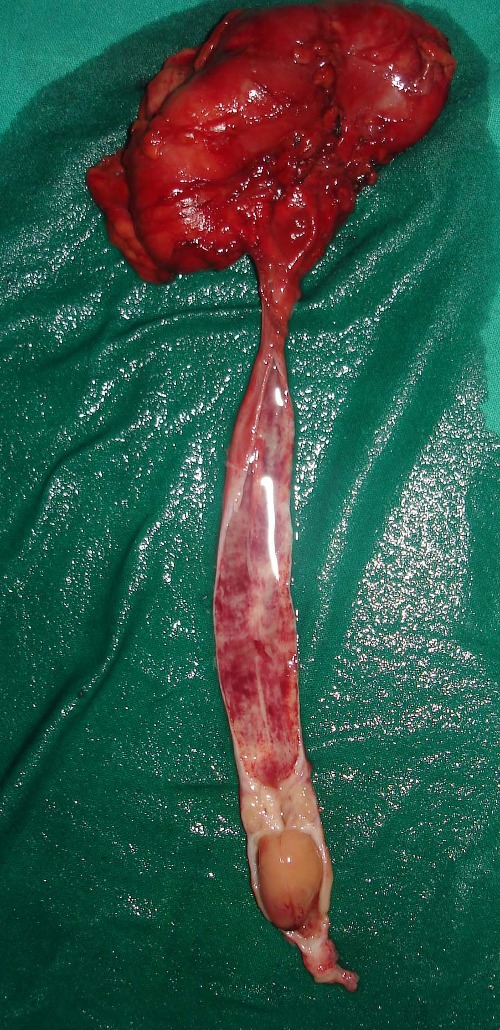
Figure 1:Longitudinal cut section of the ureter showing the polyp (1cm x1.5cm) in its lower part.

**Figure:**
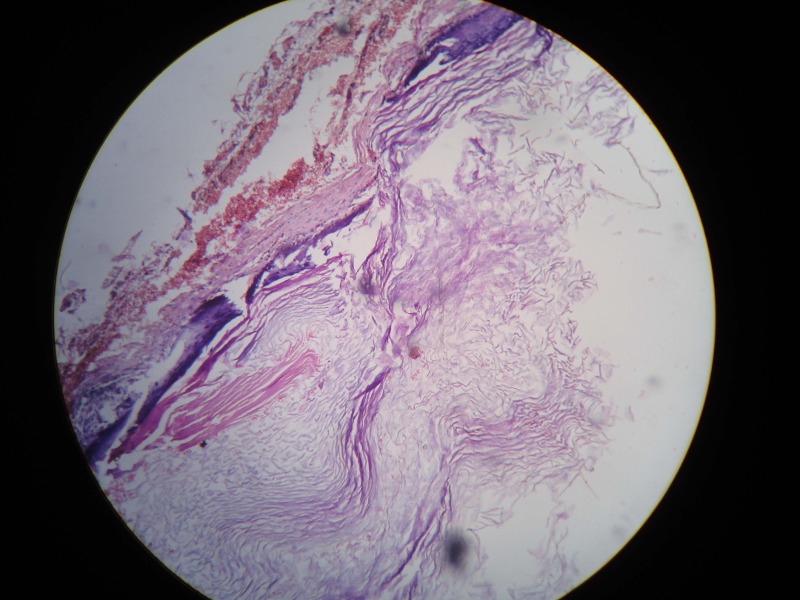
Figure 2:Histopathological examination suggests smooth muscle cells, inflammatory cells with perivascular histiocytes in a fibrovascular stroma. (H/E x 400)

## DISCUSSION

Most of the reported cases of fibroepithelial polypsare in the 3rd or 4th decade of lifeand there are few reported pediatrics patients with a mean age 9 year at presentation.[1] It occurs with almost equal frequency in both genders.[3] Though definite etiology is unknown some congenital factors in children and chronic inflammation due to UTI, calculus or DJ stent are thought to be the causative factor in adults.[3-5] Patients usually present with hematuria or flank pain.[3-6]

Ultrasonography is not a sensitive investigation to detect the ureteral lesion. Though they occur mainly in the upper ureter but sometimes they may arise in the lower ureter as in our case. In one case at cystoscopy the polyp was found prolapsing from the ureteral orifice.[7] Malignant ureteral tumors remain indistinct on conventional radiography.[5] Multidimensional CT is an effective way to detect the fibroepithelial polyp [1,4,5] The positive features in CT are continuous rim of contrast medium surrounding the solid mass, the attachment of the polyp on the ureteral wall, periureteral invasion and absence of any enlarged lymph nodes or distant metastasis.[5] Virtual CT urography is more helpful which can visualize the unique 3D morphology of ureteral fibroepithelial polyp.[5] In dilated or in obstructed urinary system noncontrast MR urography gives the similar information as IVU or CT without any contrast or any radiation exposure.[6]

Surgical intervention remained the mainstay of the management. A decade ago, the treatment was open exploration, resection of the involved ureteral segment and anastomosis.[8] In spite of preoperative knowledge of benign nature of these polyps, nephrectomy is reported in 27% of cases.[4] Nowadays the rigid or flexible ureteroscopy with accessory instruments, like basket or laser fibers or other cautery, helps in diagnosis and minimally invasive treatment of such polyps with preservation of a functioning renal unit.[1,4]

To conclude, myofibroepithelial polyp of the ureter is a rare entity. Usually patients present with hematuria of flank pain. Multidimentional CT, noncontrast MRI or ureteroscopy are the modalities to detect such lesions. In the modern era,the aim of treatment should be the renal preservation where possible.

## Footnotes

**Source of Support:** Nil

**Conflict of Interest:** None declared

